# Network Reconfiguration Among Cerebellar Visual, *and* Motor Regions Affects Movement Function *in* Spinocerebellar Ataxia Type *3*

**DOI:** 10.3389/fnagi.2022.773119

**Published:** 2022-04-11

**Authors:** Hui Chen, Limeng Dai, Yuhan Zhang, Liu Feng, Zhenzhen Jiang, Xingang Wang, Dongjing Xie, Jing Guo, Huafu Chen, Jian Wang, Chen Liu

**Affiliations:** ^1^Department of Radiology, Southwest Hospital, Third Military Medical University (Army Medical University), Chongqing, China; ^2^Department of Medical Genetics, Third Military Medical University (Army Medical University), Chongqing, China; ^3^Department of Laboratory Medicine, Southwest Hospital, Third Military Medical University (Army Medical University), Chongqing, China; ^4^Department of Neurology, Xinqiao Hospital and The Second Affiliated Hospital, Third Military Medical University (Army Medical University), Chongqing, China; ^5^Biomedical Engineering, University of Electronic Science and Technology of China, Chengdu, China

**Keywords:** spinocerebellar ataxia, SCA3, resting-state, graph theory, functional connectivity

## Abstract

**Background:**

Spinocerebellar ataxia type 3 (SCA3) is a rare movement disorder characterized with ataxia. Previous studies on movement disorders show that the whole-brain functional network tends to be more regular, and these reconfigurations correlate with genetic and clinical variables.

**Methods:**

To test whether the brain network in patients with SCA3 follows a similar reconfiguration course to other movement disorders, we recruited 41 patients with SCA3 (mean age = 40.51 ± 12.13 years; 23 male) and 41 age and sex-matched healthy individuals (age = 40.10 ± 11.56 years; 24 male). In both groups, the whole-brain network topology of resting-state functional magnetic resonance imaging (rs-fMRI) was conducted using graph theory, and the relationships among network topologies, cytosine-adenine-guanine (CAG) repeats, clinical symptoms, and functional connectivity were explored in SCA3 patients using partial correlation analysis, controlling for age and sex.

**Results:**

The brain networks tended to be more regular with a higher clustering coefficient, local efficiency, and modularity in patients with SCA3. Hubs in SCA3 patients were reorganized as the number of hubs increased in motor-related areas and decreased in cognitive areas. At the global level, small-worldness and normalized clustering coefficients were significantly positively correlated with clinical motor symptoms. At the nodal level, the clustering coefficient and local efficiency increased significantly in the visual (bilateral cuneus) and sensorimotor (right cerebellar lobules IV, V, VI) networks and decreased in the cognitive areas (right middle frontal gyrus). The clustering coefficient and local efficiency in the bilateral cuneus gyrus were negatively correlated with clinical motor symptoms. The functional connectivity between right caudate nucleus and bilateral calcarine gyrus were negatively correlated with disease duration, while connectivity between right posterior cingulum gyrus and left cerebellar lobule III, left inferior occipital gyrus and right cerebellar lobule IX was positively correlated.

**Conclusion:**

Our results demonstrate that a more regular brain network occurred in SCA3 patients, with motor and visual-related regions, such as, cerebellar lobules and cuneus gyrus, both forayed neighbor nodes as “resource predators” to compensate for normal function, with motor and visual function having the higher priority comparing with other high-order functions. This study provides new information about the neurological mechanisms underlying SCA3 network topology impairments in the resting state, which give a potential guideline for future clinical treatments.

**Clinical Trial Registration:**

[www.ClinicalTrials.gov], identifier [ChiCTR1800019901].

## Introduction

Spinocerebellar ataxia type 3 (SCA3, Machado-Joseph disease) is a rare movement disorder which caused by an abnormal expansion of the polyglutamine (polyQ) tract in the causative *ATXN3* protein (Kawaguchi et al., 1994; Rezende et al., 2018). Patients with SCA3 exhibit characteristic ataxia and nystagmus, mild cognitive (Schöls et al., 2004; Braga-Neto et al., 2012) and psychiatric symptoms (Silva et al., 2015; Yuan et al., 2019). Previous studies in SCA3 mainly focused on the changes in brain structure, such as gray matter atrophy and micro-structural white matter abnormalities (Meles et al., 2018; Guo et al., 2020; Piccinin et al., 2020). However, an increasing number of studies suggest that, in neural degeneration diseases, there is an alteration in the large-scale brain network rather than only localized dysfunction in a single brain area (Hillary and Grafman, 2017). Brain network is based on graph theory, a mathematic approach of abstract representation for large-scale brain areas, an elegant method to investigate the interaction patterns between brain areas, in which brain areas are defined as nodes and connection strengths between these areas as edges, and usually be balanced in normal optimal states (Bullmore and Sporns, 2009). It’s supposed the balance of brain network has been impaired with the significant structural atrophy in SCA3 patients, however, to date, the impact of pathological impairments on rs-fMRI brain networks in SCA3 remains elusive.

The human functional brain network usually changes to adapt to damage caused by diseases or injury. A series of network topology properties represent brain information processing, such as small-worldness, clustering coefficient, modularity, and hubs. Previous studies show that in motor disorders, the brain usually develops a more regular pattern, while the pattern is more random in cognitive and mental disorders (Zhang et al., 2011, 2019; Pereira et al., 2016; Ko et al., 2018). Increases in small-worldness and clustering coefficients have been reported in Parkinson’s disease (PD) (Berman et al., 2016; Ko et al., 2018), amyotrophic lateral sclerosis (ALS) (Zhang et al., 2019), upper limb amputation (Lyu et al., 2016), while decreased small-world index and increased global efficiency have been reported in Alzheimer’s disease (AD) (Sanz-Arigita et al., 2010; Li et al., 2016; Pereira et al., 2016), mild cognitive impairment (MCI) (Li et al., 2015) and depression (Zhang et al., 2011; Wang et al., 2016). Spared circuits and networks are usually over-engaged to maintain the efficient function of disrupted nodes to minimize behavioral deficits (Hillary and Grafman, 2017). Hubs or highly connected efficient network nodes are always centered on the optimal expression of network changes in neurodegeneration (Hillary and Grafman, 2017). For instance, striatal areas may couple with the sensorimotor cortex to offset the pathological effects in PD (Hacker et al., 2012). In multiple sclerosis, hyperconnectivity occurs between the atrophied thalamus and other regions, mainly the sensorimotor and frontal-occipital areas (Schoonheim et al., 2015). Whether the motor or visual-related brain areas, such as cerebellar, basal ganglia, cuneus and occipital lobes, corresponding to the most obvious and common clinical behavior dysfunctions in SCA3, have hyper- or hypo- connectivity with neighbors during disease progression remains unclear.

We hypothesized that brain network reorganization in patients with SCA3 is characterized by some hub nodes highly interacting with local regions and becoming a more regular network, especially in important motor- and visual-related regions. As the disease progresses, secondary hubs may be lost, causing an increase in the severity of clinical symptoms. We also hypothesized that, in patients with SCA3, brain network alterations are correlated with clinical symptoms and cognitive function.

In the current study, we examined alterations in rs-fMRI brain networks using two analytical approaches. The network topology was first examined using graph theory, which conceptualizes the connection characterizations of the brain functional network, such as global, regional network properties, and hub transfer. Furthermore, correlation analysis was used to investigate the relationships among clinical variables (disease burden, disease onset, and ataxia severity), CAG repeats, functional connectivity, and network topological attributes.

## Materials and Methods

### Participants

In this study, 46 patients with confirmed diagnosis of SCA3 and 43 age- and sex-matched healthy individuals were recruited *via* telephone follow-up care and advertisements on apps and searched in a big-data intelligence database called Yiducloud Technology, at First Affiliated Hospital, Army Medical University. Genetic testing for CAG expansion using peripheral blood samples was conducted at the Genetic Education and Research Laboratory of Army Medical University. The CAG repeats in patients with SCA3 ranged from 57 to 72 (Table 1). None of the patients received systematic or regular medical treatment. Exclusion criteria for patients and healthy participants included any brain stimulation, brain surgery, other genetic disease, an untreated psychiatric condition, current or past neurological or psychiatric disorders, left-handedness, or any contraindications to MRI. We excluded three SCA3 patients and one healthy participant for their left-handedness, two SCA3 patients, and one healthy participant for large head motion. Written informed consents were obtained from all participants. The First Affiliated Hospital of the Army Medical University Review Board approved this study.

**TABLE 1:** Demographic, clinical, and cognitive variables.

Variable	SCA3 (*n* = 41)	HCs (*n* = 41)	T/W/x^2^ (*p* Value)
Gender (M/F)	23/18	24/17	2.49(0.35^*b*^)
Age (years)	17–67 (40.51 ± 12.13)	21–64 (40.10 ± 11.56)	−0.23(0.82^*a*^)
Onset age (years)	18–58 (35.25 ± 10.67)		
Disease duration	0–21 (6.76 ± 4.98)		
CAG repeats	57–72 (65.71 ± 3.57)		
SARA	0–33 (10.62 ± 8.54)		
ICARS	0–73 (27.33 ± 19.43)		
ICARS—posture and gait disturbances	0–34 (12.78 ± 9.34)		
ICARS—kinetic functions	0–50 (12.76 ± 10.40)		
ICARS—dysarthria	0–8 (1.71 ± 2.00)		
ICARS—oculomotor disorders	0–6 (1.80 ± 1.79)		
HMAD	0–32 (7.157 ± 6.898)	0–17 (1.73 ± 3.39)	−4.52(0.00^***a^)
MoCA	10–30 (22.95 ± 4.96)	19–30 (27.37 ± 2.93)	4.91(0.00^***a^)
RVR	16–72 (36.53 ± 10.93)	32–80 (50.83 ± 11.51)	5.77(0.00^***a^)
DS	3–13 (8.72 ± 2.13)	5–16 (9.51 ± 2.16)	1.68(0.10^*a*^)
ADL+IADL	20–66 (28.39 ± 12.94)	20–21 (20.02 ± 0.16)	−5.97(0.00^***c^)
MMSE	13–30 (27.28 ± 3.47)	22–30 (28.81 ± 1.81)	2.60(0.01^**c^)

*Values are presented as a range (mean ± standard deviation).*

*Onset age, age at onset of ataxia symptoms; Duration: Duration between onset and examination age; SARA, Scale for the Assessment and Rating of Ataxia; ICARS, International Cooperative Ataxia Rating Scale; ADL, Activities of Daily Living; IADL, Instrumental Activities of Daily Living; MMSE, Mini-Mental State Examination; MoCA, Montreal Cognitive Assessment; RVR, rapid verbal retrieval; DS, Digit Span; HAMD, Hamilton Rating Scale for Depression; M/F, Males/Females.*

**, P < 0.01.

***, P < 0.001.

*a, Two-sample two-tailed t test.*

*b, Two-tailed Pearson chi-square test.*

*c, Mann–Whitney U-test.*

### Clinical and Cognitive Variables

The neurological motor functions were evaluated with scales including the International Cooperative Ataxia Rating Scale (ICARS) (Trouillas et al., 1997), the Scale for Assessment and Rating of Ataxia (SARA) (Schmitz-Hubsch et al., 2006) and the activities of daily living (ADL) and instrumental activities of daily living (IADL) for daily physical functioning (Ahrenfeldt et al., 2018). For cognitive assessment, we used the Mini-Mental State Examination (MMSE) and Montreal Cognitive Assessment (MoCA) to assess the global cognitive function (Folstein et al., 1975); the Digit Span test (Blackburn and Benton, 1957) for verbal working memory; Rapid Verbal Retrieval (RVR) for Category fluency (Lucas et al., 1998). For emotional assessment, the Hamilton Rating Scale for Depression-24 (HAMD-24) were used to rate the severity of depressive symptoms (Kovacs et al., 1981). ICARS and SARA were rated only in SCA3 patients.

### Magnetic Resonance Imaging Data Acquisition

We performed MRI scanning on a 3.0T scanner (Siemens Tim Trio, Germany) using an 8-channel receiver phased-array head coil at the First Affiliated Hospital, Army Medical University. During the MRI study, the study participants were placed in the supine position with their eyes closed and awake, and foam pads were secured around their heads to minimize head movement. The parameters of the gradient-echo EPI sequence were as follows: 240 volumes, repetition time = 2,000 ms, echo time = 30 ms, 36 slices, voxel size = 3 mm × 3 mm × 3.99 mm. For registration, we collected a high-resolution structural image using spoiled gradient echo pulse sequence [echo time = 2.52 ms, repetition time = 1,900 ms, flip angle = 9°, field of view (FOV) = 256 × 256, inversion time = 900 ms, slice thickness = 1 mm, contiguous axial slices = 176, voxel size = 1 mm × 1 mm × 1 mm].

### Magnetic Resonance Imaging Data Preprocessing

Resting-state data preprocessing was conducted using DPARSF (Chao-Gan and Yu-Feng, 2010) and SPM12^1^. The first five timepoints were discarded for each participant for magnetization equilibration. Slice-timing, head-motion correction was applied on the remaining 235 volumes. Participants in both groups were excluded if their maximum displacements were greater than 2 mm or if their head rotations were greater than 2°. Then, the images were intensity normalized and resampled to a 3-mm isotropic voxel. As suggested in previous studies, spatial smoothing was not applied due to avoid introducing artificial local spatial correlations (Lin et al., 2015). Finally, a temporal filter was applied to focus on low-frequency fluctuations (0.01–0.1 Hz).

### The Whole-Brain Functional Connectivity Network

#### Network Construction

We used the Conn Toolbox to analyze the preprocessed data (Rubinov and Sporns, 2010). A nuisance regression was performed with a method aCompCor method which contained six subject-specific realignment parameters, controlling for the signals from white matter and CSF. According to the automated anatomical labeling (AAL) atlas, we defined region of interests (ROIs) by dividing the whole brain area into 116 cortical and subcortical regions (Tzourio-Mazoyer et al., 2002), extracted time series from these 116 regions, computed the temporal correlations between all possible pairs of regions, normalized the correlation coefficient using Fisher’s r-to-z transformation, finally constructed a 116 × 116 correlation matrix for each participant.

#### Network Analysis

We performed network and statistical analyses using the Graph Analysis Toolbox (GAT) (Hosseini et al., 2012). To avoid a single arbitrary threshold and reduce the number of comparisons across thresholds, we chose a range of density thresholds and the integral over this range (Hosseini and Kesler, 2013). The density thresholds were set from 0.3 to 0.45 in 0.01 steps. Under the lower bound of cost of 0.3, both groups were not fragmented, as estimated by GAT. Wiring cost above 0.45 is the upper limit for brain networks, leading to more random networks and a smaller world (Meng et al., 2014). In each subject’s functional network, the underlying topological organization was investigated using unweighted binary adjacency matrices to eliminate the interference of the changes in absolute connectivity. If the element *z*_*ij*_ of the functional connectivity matrix was greater than a threshold, the corresponding element of the binarized network matrix was set to 1; otherwise, it was set to 0.

We calculated the following brain network topological parameters: (small-worldness (σ), clustering coefficient (*C_p*), shortest path length (*L_p*), normalized clustering coefficient (γ), characteristic path length (λ), local efficiencies (*E*_*Loc*_), modularity, and nodal parameters (including node clustering coefficient, node efficiency, and hubs). These parameters determine the brain network information processing patterns and are usually anomalous in neurodegenerative diseases (Watts and Strogatz, 1998). Small-worldness is an index represents a balanced network integration and segregation. The clustering coefficient represents the degree of network segregation of a node, for a given node, it means the proportion of this node’s neighbors that are also neighbors with each other. The shortest path length of two nodes is the minimum number of edges between them. The characteristic path length is a property reflects network integration, which equals the average shortest path length between each pair of nodes in this network. The local efficiency of a network is the average of the local efficiencies across all nodes. Modularity measures the degree how many subnetworks (modules) a network can be divided, maximal intra-module connections and minimal inter-module connections were coexisting in each module (Zhang et al., 2019). The nodal clustering coefficient is defined as the proportion of the existing edges to all possible edges of this node. The nodal local efficiency is the global efficiency of a subgraph contains the nearest neighbors of the node (Zhang et al., 2019). In our study, a hub is defined as a node which degree was above the mean network degree at least one standard deviation (Bruno et al., 2012). According to the main concerns in clinical behavior symptoms, we defined primary hubs to be motor or visual related hubs, while secondary hubs to be other functional hubs. Additionally, the area under the curve (AUC) was calculated within the sparsity range for a summarized scalar for topological properties and avoiding the specific threshold selection, and compared the results between healthy controls (HCs) and patients with SCA3 (Tu et al., 2019).

### Statistical Analysis

In the brain network topological analysis, a group comparison was conducted for each density using a permutation test (1,000 iterations; *P* < 0.05), controlling age, sex, and total gray matter volume. The total gray matter volume was included to focus on the changes in the whole-brain network functional integration, which were independent of the structural changes (Meng et al., 2014). The false discovery rate for multiple comparisons (FDR, *p* < 0.05) was applied for the global metric results correction, 95% confidence interval (95% CI) was also reported.

To understand the relationships between the resting-state functional connectivity (rsFC), global network measures, demographics, CAG repeats, and clinical and cognitive variables, their correlation coefficients were calculated using a two-tailed correlation analysis. At the nodal level, correlations in only significantly different brain areas were examined. Cognitive scores were normalized (mean and standard deviation). Five patients with SCA3 were asymptomatic and thus excluded from the correlation analysis due to lack of data on disease duration and clinical symptoms.

We assessed the normality of the continuous variables using the Kolmogorov-Smirnov test. The scores of ICARS-oculomotor disorders, ADL+IADL, and MMSE scores were not normally distributed even with logarithmic transformation (Supplementary Table 1). All comparisons of averages were performed using two-sample *t*-tests between the SCA3 and the control group, except for sex and non-normal score comparisons. The group difference of sex was conducted using a chi-square test, while non-normal score was conducted using the Mann–Whitney *U*-test. Pearson’s correlation was applied for normally distributed variables and Spearman’s rank correlation for non-normally distributed variables, with age and sex as covariates. The false discovery rate (FDR) method was applied to all correlation analyses, with a corrected significance level of *P*_*FDR*_ < 0.05. 95% CI in correlational analysis was obtained using transformation between r and Z.

## Results

### Clinical Testing

In all, 41 patients with SCA3 (18 female; age: 40.51 ± 12.13 years) and 41 age- and sex-matched HCs (17 female, age: 40.10 ± 11.56 years) were considered. No significant differences in age or sex were found between the two groups (T = −0.23, *P* = 0.82 and *x*^2^ = 2.49, *P* = 0.35, respectively). Demographic, clinical, and cognitive variables are summarized in Table 1.

In cognition, significant group differences were found for all cognitive tasks except the digit span task (Table 1). Patients with SCA3 had significant worse performance than HCs.

### Network Topology

#### Global Network Measures Differences

**FIGURE 1:**
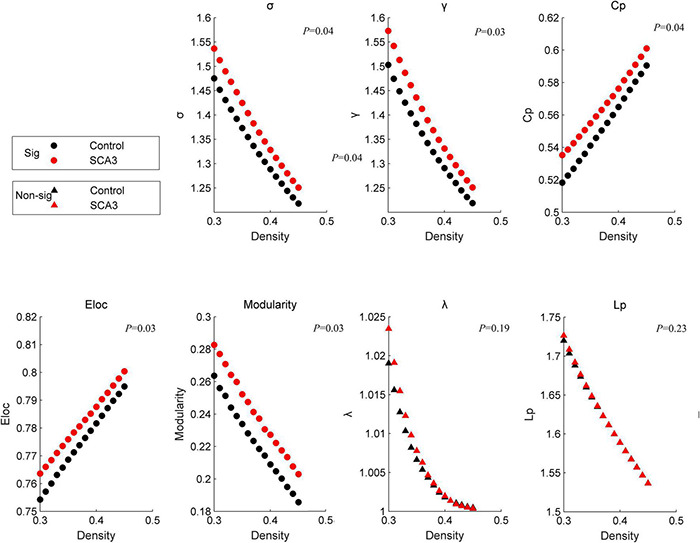
Disrupted graphic topology properties. Dots represent significant differences between the two groups. Triangles represent no significant differences between the two groups. Black/NC represents normal control subjects. Red/SCA3, represents SCA3 patients. σ, small-worldness; *C_p*, clustering coefficient; γ, normalized clustering coefficient;*L_p_*, shortest path length;λ, the characteristic path length; *E*_*Loc*_, local efficiency;*E_glob_*, global efficiency; *T*, transitivity;α, assortativity.

At a sparsity range of 0.30–0.45, both groups had small-world regimes (σ > 1.220). Global network measures between the two groups significantly increased in patients with SCA3 compared to HCs (Figure 1), σ[*P* = 0.039, 95% CI (−0.061, −0.002)], γ [*P* = 0.031, 95% CI (−0.064, −0.003)], modularity [*P* = 0.017, 95% CI (−0.027, −0.003)], *E*_*loc*_ [*P* = 0.029, 95% CI (−0.011, −0.001)], and *C_P* [*P* = 0.037, 95% CI (−0.022, −0.008)], while the other topology parameters, including λ and *L_P* had no significant differences.

**FIGURE 2:**
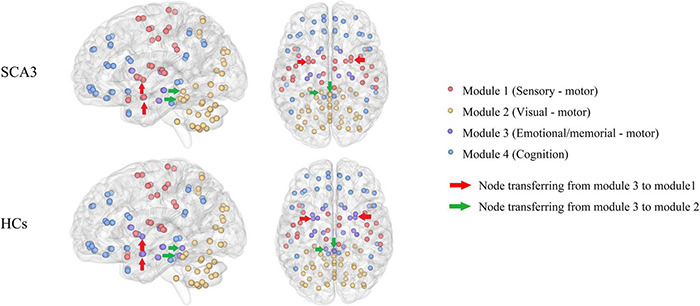
Brain network modularity in patients with SCA3 and HCs. Nodes of the same color are in the same module. Different colors represent different modules. The red arrows indicate the nodes transferred from emotional/memorial—motor into sensory—motor modularity in SCA3 compared to HCs, including the bilateral amygdala and bilateral putamen. Green arrows indicate the nodes transferred from emotional/memorial—motor into visual—motor modularity in SCA3 compared to HCs, including bilateral cerebellar lobules III and vermis I, II, III. HC, healthy controls; SCA3, spinocerebellar ataxia 3.

Patients with SCA3 had different brain modules compared with healthy controls. Some nodes in the memorial -, emotional-, and motor-related modules in HCs were transferred into other modularity in SCA3, such as the bilateral amygdala and bilateral putamen were transferred into the sensorimotor network in the SCA3 group, while the bilateral cerebellum lobule III and vermis I, II, and III were transferred into the visual network (Figure 2).

#### Regional Network Measures Differences

**FIGURE 3:**
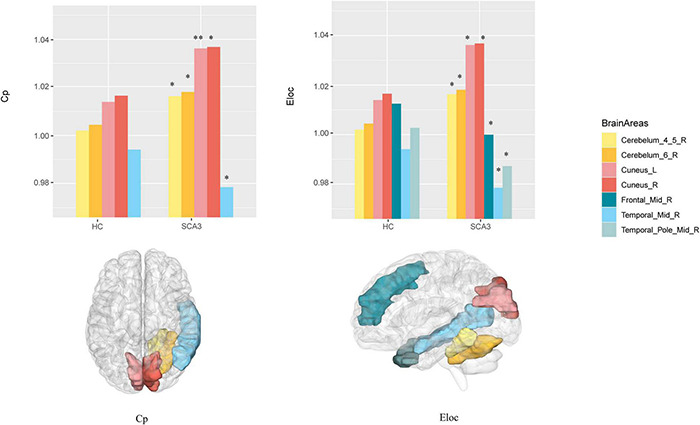
Brain Areas that significantly differ in nodal properties between the two groups. Cerebellum_4_5_R, right cerebellar lobule IV–V; Cerebellum_6_R, right cerebellar lobule VI; Cuneus_L, left cuneus; Cuneus_R, right cuneus; Frontal _Mid_R, right middle frontal lobe; Temporal_Mid_R, right middle temporal lobe; Temporal_Pole_Mid_R, right middle temporal pole; The *P* -values were corrected using FDR correction. Warm colors (yellow, orange, pink, red) represent an increased index, while cold colors (green, blue) represent a decreased index in SCA3 compared to HC. *, *P*_*FDR*_ < 0.05; ^**^, *P*_*FDR*_ < 0.01.

Compared with HCs, the node clustering coefficient in patients with SCA3 decreased in the right middle frontal gyrus, and increased in the bilateral cuneus, right cerebellar lobule VI, and right cerebellar lobules IV and V. The local efficiency in patients with SCA3 decreased in the right middle frontal gyrus and the right middle temporal gyrus, and increased in the bilateral cuneus gyrus, right cerebellar lobules IV and V, and right cerebellar lobule VI (Figure 3).

#### Network Hubs

**FIGURE 4:**
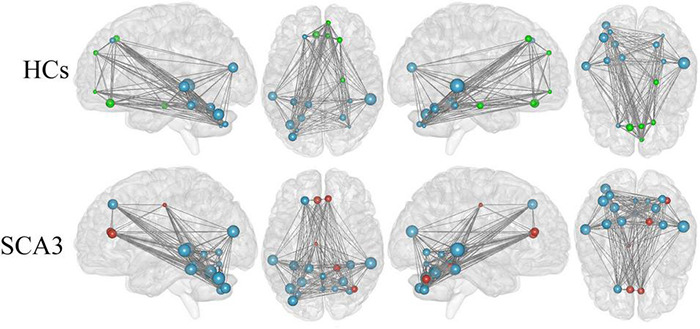
Hub regions identified by nodal degree in the HCs and patients with SCA3. The node sizes indicate their relative degree (*D*_*nod*_). A node was identified as a hub if its normalized nodal degree was higher than 1 SD of all the nodes of the network. Red nodes represent the hubs identified only in the SCA3 group. Green nodes represent the hubs identified only in the HC group. Blue nodes represent the hubs identified in both groups. HC, healthy controls; SCA3, spinocerebellar ataxia 3.

We render the network hubs for both groups in Figure 4. Patients with SCA3 and HCs were similar in most network hub locations. The hubs were identified for both groups, including the frontal, fusiform, occipital, temporal, and cerebellar regions. Additional hubs were identified for the SCA3 group in the bilateral anterior cingulum gyrus, left middle cingulum gyrus, right lingual gyrus, lobules IV and V, vermis VI, right cerebellar lobules IV and V, and right cerebellum crus I. In HCs, additional hubs were distributed in the right orbital frontal gyrus, right superior medial frontal gyrus, right superior frontal gyrus, right parahippocampal cortex, and bilateral rectus gyrus (Supplementary Table 2).

### Correlations

#### Relationship Between Genetic, Clinical, and Cognitive Variables in Patients With SCA3

Correlation analyses (adjusted for age and sex) showed that CAG repeats were not significantly correlated with any clinical and cognitive variables after false discovery rate (FDR) multiple comparison correction. The details are listed in Supplementary Table 3.

#### Relationship Between Clinical Variables and Network Measures in Patients With SCA3

At the global level (Table 2), we found that σ and γ were significantly correlated with ICARS dysarthria and ADL+IADL. Modularity was significantly correlated with ADL+IADL.

**TABLE 2:** Correlations between global neuroimaging attributions and clinical variables.

Variable	σ	C_P_	γ	E_l*oc*_	Modularity	λ	L_P_
Onset age (years) (P)	−0.16 (*0.44*) [−0.47, 0.18]	−0.03 (*0.84*) [−0.36, 0.30]	−0.15 (*0.48*) [−0.46, 0.19]	−0.03 (*0.87*) [−0.35, 0.30]	−0.17 (*0.40*) [−0.47, 0.17]	0.03 (*0.85*) [−0.30, 0.36]	0.03 (*0.92*) [−0.31, 0.35]
Disease duration (P)	0.16 (*0.44*) [−0.18, 0.47]	0.03 (*0.84*) [−0.30, 0.36]	0.15 (*0.48*) [−0.19, 0.46]	0.03 (*0.87*) [−0.30, 0.35]	0.17 (*0.40*) [−0.17, 0.47]	−0.03 (*0.85*) [−0.36, 0.30]	−0.03 (*0.92*) [−0.35, 0.31]
CAG repeats (P)	0.24 (*0.23*) [−0.09, 0.53]	0.14 (*0.59*) [−0.19, 0.45]	0.24 (*0.23*) [−0.09, 0.53]	0.14 (*0.59*) [−0.20, 0.45]	0.25 (*0.27*) [−0.09, 0.53]	0.10 (*0.69*) [−0.24, 0.42]	0.11 (*0.65*) [−0.23, 0.42]
SARA (P)	0.44 (*0.03*) * [0.13, 0.67]	0.29 (*0.25*) [−0.05, 0.56]	0.44 (*0.03*) * [0.16, 0.67]	0.29 (*0.24*) [−0.04, 0.56]	0.35 (*0.14*) [0.02, 0.61]	0.22 (*0.55*) [−0.11, 0.52]	0.22 (*0.53*) [−0.11, 0.52]
ICARS (P)	0.35 (*0.08*) [0.04, 0.62]	0.28 (*0.25*) [−0.05, 0.56]	0.36 (*0.07*) [0.04, 0.62]	0.28 (*0.24*) [−0.05, 0.56]	0.30 (*0.19*) [−0.03, 0.57]	0.22 (*0.55*) [−0.11, 0.52]	0.22 (*0.53*) [−0.11, 0.52]
ICARS—posture and gait disturbances (P)	0.36 (*0.08*) [0.04, 0.62]	0.29 (*0.25*) [−0.04, 0.57]	0.36 (*0.07*) [0.04, 0.62]	0.29 (*0.25*) [−0.04, 0.57]	0.33 (*0.17*) [−0.003, 0.59]	0.23 (*0.55*) [−0.11, 0.52]	0.22 (*0.53*) [−0.11, 0.52]
ICARS—kinetic functions (P)	0.32 (*0.12*) [−0.01, 0.59]	0.25 (*0.34*) [−0.09, 0.53]	0.32 (*0.12*) [−0.01, 0.59]	0.25 (*0.31*) [−0.09, 0.53]	0.27 (*0.23*) [−0.07, 0.55]	0.18 (*0.55*) [−0.16, 0.48]	0.18 (*0.53*) [−0.15, 0.48]
ICARS—dysarthria (P)	0.41 (*0.04*) * [0.10, 0.65]	0.29 (*0.25*) [−0.05, 0.56]	0.42 (*0.04*) * [0.10, 0.66]	0.29 (*0.25*) [−0.04, 0.56]	0.28 (*0.23*) [−0.05, 0.55]	0.25 (*0.55*) [−0.08, 0.54]	0.25 (*0.53*) [−0.09, 0.53]
ICARS—oculomotor disorders (S)	0.23 (*0.23*) [−0.09, 0.53]	0.08 (*0.73*) [−0.25, 0.40]	0.23 (*0.18*) [−0.08, 0.53]	0.10 (*0.65*) [−0.24, 0.41]	0.19 (*0.36*) [−0.14, 0.50]	0.06 (*0.82*) [−0.27, 0.38]	0.02 (*0.93*) [−0.31, 0.34]
HMAD (P)	0.44 (*0.03*) * [0.14, 0.68]	0.35 (*0.25*) [0.02, 0.61]	0.44 (*0.03*) * [0.13, 0.67]	0.35 (*0.24*) [0.02, 0.61]	0.40 (*0.08*) [0.08, 0.64]	0.18 (*0.55*) [−0.16, 0.48]	0.18 (*0.53*) [−0.15, 0.48]
ADL+IADL (S)	0.63 (*0.00*) ** [0.38, 0.79]	0.30 (*0.25*) [−0.02, 0.57]	0.44 (*0.01*) ** [0.37, 0.79]	0.31 (*0.25*) [−0.02, 0.58]	0.57 (*0.01*) ** [0.30, 0.76]	0.24 (*0.55*) [−0.10, 0.53]	0.21 (*0.53*) [−0.13, 0.50]
MMSE (S)	−0.28 (*0.18*) [−0.56, 0.05]	−0.21 (*0.38*) [−0.51, 0.12]	−0.25 (*0.15*) [−0.55, 0.07]	−0.24 (*0.32*) [−0.53, 0.09]	−0.23 (*0.28*) [−0.52, 0.10]	−0.22 (*0.55*) [−0.51, 0.11]	−0.22 (*0.53*) [−0.52, 0.11]
MoCA (P)	−0.12 (*0.52*) [−0.43, 0.21]	−0.13 (*0.62*) [−0.44, 0.21]	−0.13 (*0.49*) [−0.44, 0.21]	−0.13 (*0.62*) [−0.44, 0.21]	−0.11 (*0.56*) [−0.42, 0.23]	−0.16 (*0.55*) [−0.47, 0.18]	−0.17 (*0.53*) [−0.47, 0.17]
RVR (P)	−0.09 (*0.62*) [−0.4, 0.24]	−0.11 (*0.63*) [−0.43, 0.22]	−0.09 (*0.59*) [−0.41, 0.24]	−0.11 (*0.63*) [−0.43, 0.22]	−0.08 (*0.66*) [−0.40, 0.26]	−0.11 (*0.69*) [−0.42, 0.23]	−0.12 (*0.65*) [−0.43, 0.22]
DS (P)	−0.13 (*0.52*) [−0.44, 0.21]	−0.21 (*0.38*) [−0.51, 0.12]	−0.14 (*0.49*) [−0.45, 0.20]	−0.22 (*0.35*) [−0.51, 0.11]	−0.12 (*0.54*) [−0.44, 0.21]	−0.21 (*0.55*) [−0.50, 0.13]	−0.20 (*0.53*) [−0.50, 0.14]

*The values are presented as correlation coefficients with r (P_FDR_) [95% confidence interval].*

*Onset age, age at onset of ataxia symptoms; Duration, Duration between onset and examination age; SARA, Scale for the assessment and rating of ataxia; ICARS, International Cooperative Ataxia Rating Scale; ADL, Activities of Daily Living; IADL, Instrumental Activities of Daily Living; MMSE, Mini-Mental State Examination; MoCA, Montreal Cognitive Assessment; RVR, rapid verbal retrieval; DS, Digit Span; HAMD, Hamilton Rating Scale for Depression; σ, small-worldness; γ, Normalized clustering coefficient.*

*, P_FDR_ ≤ 0.05; **, P_FDR_ ≤ 0.01.

*P, Pearson correlation.*

*S, Spearman rank correlation. The meaning of the bold values is to make the significant results more obvious to readers.*

At the nodal level (Table 3), the correlation analysis demonstrated that in the left cuneus, *C_P* was negatively correlated with ADL + IADL [*r* = −0.48, *P*_*FDR*_ = 0.02, 95% CI (−0.70, −0.17)] and ICARS dysarthria [*r* = −0.42, *P*_*FDR*_ = 0.03, 95% CI (−0.66, −0.11)]; *E*_*loc*_ was negatively correlated with ADL+IADL [*r* = −0.49, *P*_*FDR*_ = 0.02, 95% CI (−0.70, −0.19)] and ICARS dysarthria [*r* = −0.41, *P*_*FDR*_ = 0.05, 95% CI (−0.65, −0.10)]; In the right cuneus, *C_P* and *E*_*loc*_was negatively correlated with ICARS dysarthria (*r* = −0.42, *P*_*FDR*_ = 0.04, 95% CI [−0.67, −0.12], *r* = −0.43, *P*_*FDR*_ = 0.05, 95% CI [−0.66, −0.11], respectively).

**TABLE 3:** Correlations between regional neuroimaging attributions and clinical variables.

Variable	Left cuneus		Right cuneus	
	Cp	Eloc	Cp	Eloc
ICARS-Dysarthria (P)	−0.42 *(0.03)* * [−0.66, −0.11]	−0.41 *(0.05)* * [−0.65, −0.10]	−0.43 *(0.03)* * [−0.67, −0.12]	−0.43 *(0.05)* * [−0.66, −0.11]
ADL+IADL (S)	−0.48 *(0.02)* * [−0.70, −0.17]	−0.49 *(0.02)* * [−0.70, −0.19]	−0.26 *(0.34)* [−0.54, 0.07]	−0.25 *(0.39)* [−0.54, 0.08]

*The values are presented as correlation coefficients with r (P_FDR_) [95% confidence interval].*

ICARS, International Cooperative Ataxia Rating Scale; ADL, Activities of daily living; IADL, Instrumental Activities of Daily Living; Cp, Clustering coefficient; Eloc, Local efficiency; *, P_FDR_ ≤ 0.05.

*(P), Pearson correlation.*

*(S), Spearman rank correlation. The meaning of the bold values is to make the significant results more obvious to readers.*

#### Relationship Between Connectivity Strength and Clinical Symptoms in Patients With SCA3

**FIGURE 5:**
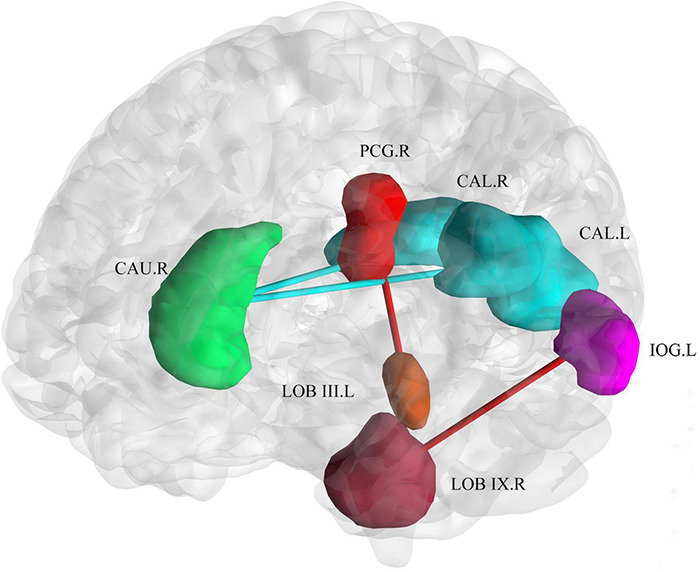
Correlation of connectivity change with disease duration and age of onset. The plot shows that with an increase in duration or earlier age of onset, the functional connectivity strength between the bilateral calcarine and the right caudate decreased. The functional connectivity strength between the inferior occipital gyrus and right cerebellar lobule IX, and the right posterior cingulum gyrus and left cerebellar lobule III increased. Cold colors (blue and green) represent decreased functional connectivity. Warm colors (red and brown) represent increased functional connectivity. CAU.R, caudate nucleus; PCG.R, right posterior cingulum gyrus; CAL.R, right calcarine gyrus; CAL.L, left calcarine gyrus; IOG.L, left inferior occipital gyrus; LOB III. L, left cerebellar lobule III; LOB IX.R, right cerebellar lobule IX.

With respect to the brain functional changes, functional connectivity strength was significant correlate with disease duration including the right posterior cingulum gyrus and left cerebellar lobule III [*r* = 0.73,*P_FDR_* = 0.01, 95% CI (0.53, 0.86)], left inferior occipital gyrus and right cerebellar lobule IX [*r* = 0.71,*P_FDR_* = 0.01, 95% CI (0.50, 0.84)], left calcarine and right caudate nucleus [*r* = −0.66, *P*_*FDR*_ = 0.04, 95% CI (−0.81, −0.43)], right calcarine and right caudate nucleus [*r* = −0.65, *P*_*FDR*_ = 0.05, 95% CI (0.41, 0.81)] (Figure 5). The relationship between onset age and functional connectivity in these regions had similar correlation strengths but opposite directions.

## Discussion

In this study, we explored the large-scale network reconfiguration in patients with SCA3. Compared to healthy controls, the patients with SCA3 have the increase in topological specialization with higher small-worldness, clustering coefficient, modularity, and local efficiency, indicating a more regular network that is more energy-costing and metabolically active in information processing at core nodes (Ko et al., 2018). Modularity shifts and hub transfers were found with changes in topological attributions in several brain regions, reflecting disequilibrium between global integration and local segregation in the SCA3 brain network, which may underlie motor and visual impairments (Zhang et al., 2019). Several network attributions were positively correlated with symptom severity, indicating that increased specialization might represent a compensatory mechanism that offsets clinical severity. Meanwhile, network specialization increased, but was inversely associated with symptom severity in the bilateral cuneus, suggesting a visual pathological process. Furthermore, we showed debilitated cortico-cortical and strengthened cortico-cerebellar functional connectivity with longer disease duration and earlier age onset. We did not find significant relationships between CAG repeat length and clinical behavior or brain network profiles. We also demonstrate that the brain network of patients with SCA3 shifts to be more regular, and this tendency is correlated with clinical severity but has no relationship with gene burden.

The brain network in patients with SCA3 became more specialized and shifted into a more regular network with increased small-worldness, clustering coefficient, local efficiency, and modularity (Figure 1). The brain network has a small-world organization indexing the balance between network integration and segregation (Watts and Strogatz, 1998). The results in this study indicate an occurrence of a less optimal topological architecture in the SCA3 brain functional networks. Similar changes have been associated with the general patterns of several neuropsychiatric diseases, such as multiple sclerosis (MS) (Gamboa et al., 2014; Koubiyr et al., 2019), PD (Ko et al., 2018), and ALS (Zhang et al., 2019). These changes were diffusely mediated across multiple subsystems, including the bilateral cuneus gyrus and cerebellar regions (Figure 3), which are important regions for visual and motor functions (Hernandez-Castillo et al., 2015; Rezende et al., 2018). Network-level connectivity often occurs with modular shifts and hub transfer to adapt to the local pathological changes in neurodegenerative disorders (Zhang et al., 2019). The nodes belonging to the subcortical network in the healthy group were transferred into the somatosensory motor and posterior network in patients with SCA3. The brain areas which were found abnormal in this study are consistent with the findings reported in previous structural studies, include cerebellar lobules, vermis, putamen and cuneus gyrus (Meles et al., 2018). These changes indicate that the visual and motor-related regions, involved in cuneus, cerebellar regions, became more important and interconnected with the neighbors more intensely, while the brain regions, for example, left middle cingulum gyrus, right lingual gyrus and right orbital frontal gyrus and right parahippocampal cortex, etc., related to communication, memory, attention, and executive functions lost their power. Visual and motor-related regions act like the “resource predator” to compensate for gait ataxia and oculomotor dysfunction in patients with SCA3. The hyperconnectivity phenomenon commonly occurs in brain imaging studies with injured human function, which implies a trade-off in most of the central and metabolically efficient areas (Hillary and Grafman, 2017).

Verifying whether the changes in neurodegeneration are compensatory or pathological is not trivial (Shine et al., 2019). The global and local integration attributions, such as σ and, λ were positively associated with motor symptom severity measured by SARA, ICARS-dysarthria, ADL + IADL (Table 2), and the *E*_*loc*_ was significantly higher in patients with SCA3. However, the nodal clustering coefficient and local efficiency were inversely correlated with the ataxia severity in the bilateral cuneus gyrus (Table 3). Thus, a contradiction occurs. The more the global network tended to be a regular network, the more severe the symptoms and the higher the network parameters, particularly in the motor-related regions, while the other secondary regions had lower network properties. In contrast, if it is a pathological mechanism, the network parameters would be higher in the motor areas, such as the cerebellum, superior motor area, or basal ganglia (Taroni and DiDonato, 2004). However, at the nodal level, no motor-related areas were found to be positively correlated with the clinical symptoms; therefore, the cuneus, a visual-related area, negatively correlated with the clinical symptoms, reflecting that the global brain network alterations in the SCA3 patients were a compensatory process (Shine et al., 2019). This suggests that the local integration that occurred in the cuneus gyrus might help maintain motor function at the sacrifice of the visual function, which represents a compensatory mechanism (O’Callaghan et al., 2016).

Structural brain lesions cause widespread reconfigurations in the functional network, represented by large-scale topology and functional connectivity (Zhang et al., 2019). As mentioned above, we demonstrated that patients with SCA3 showed abnormal topological attributions in a large-scale functional network. In this study, functional connectivity between the right caudate nucleus and the bilateral calcarine gyrus was found to be negatively correlated with disease duration, while the functional connectivity between the cerebellum and right posterior cingulate gyrus/left inferior occipital gyrus was positively associated. The diminished connectivity of the caudate-cortical circuits in the patients with SCA3 was most likely a consequence of basal ganglia dysfunction, which in turn might have led to a deficiency in the visual-motor function in patients with SCA3 patients (Hanssen et al., 2018). In previous studies, an increase in functional connectivity (FC) might compensate the structurally atrophied brain regions to maintain their function, and this positive correlation between disease duration and cerebellar-cortical functional connectivity might reflect a compensatory mechanism (Hernandez-Castillo et al., 2015). The cortico-striatum and cortico-cerebellar abnormalities underscore intrinsic extrapyramidal involvement in patients with SCA3 patients (Meles et al., 2018). In PD, the effective connectivity of the striatum–cortex is weakened, whereas the cortico-cerebellar connectivity is strengthened during self-initiated movement (Wu et al., 2011). Hyperconnectivity may sustain information communication between nodes to minimize behavioral deficits (Hillary and Grafman, 2017).

No significant relationships were found between CAG repeat length and clinical behavior or brain network profiles in this study. In previous SCA3 studies, CAG repeats were significantly correlated with gray matter atrophy of the cerebellar culmen, brainstem, and pons (Peng et al., 2019), whereas other studies failed to find such correlations (Huang et al., 2017). These discrepancies might be due to potential confounding factors, such as disease progression, suggesting further studies with a larger cohort.

## Limitations

There are several potential confounding variables to be addressed. First, significant visual-related brain areas were identified in our study. However, we did not check the visual ability or design a visual-related fMRI task to investigate this issue in depth. Another limitation is that the network node choice was slightly arbitrary. We parcellated the whole brain into 116 regions according to the AAL atlas, which might induce considerable variations in the graph-based analysis; other parcellation methods might be compared in future studies. Third, the high threshold (FDR, *p* < 0.05) might increase the possibility of false positive *p* values in results, we should find ways to accumulate more SCA3 patients, e.g., multi-center cooperation, to improve the statistic power.

## Conclusion

The current study demonstrates that global functional brain network regularization occurred in SCA3 patients with increasing small-worldness, clustering coefficient, and local efficiency. At the nodal level, motor and visual related regions had increased clustering coefficient and local efficiency, while cognitive and emotional regions had a decrease in these factors, just like motor and visual regions recruited adjacent nodes to maintain normal functions as “resource predators.” These alterations were correlated with the ataxia severity. In functional connectivity analysis, we found that functional connectivity strength changed in the cortico-cortical and cortical-cerebellum circuits as the disease progressed, and that this compensation mechanism had hierarchy; motor regions had the highest priority while visual regions were secondary. Our results support the hypothesis that specific cerebrocerebellar network reorganization occurs with disease progression and clinical symptoms in SCA3. Collectively, according to our findings, we cloud use some invasive techniques in future clinical treatment, such as repeat transcranial magnetic stimulation (rTMS), transcranial direct current stimulation (TDCs), which would modulate the targeted neuron population activity, to enhance or depress the function of these brain areas in large-scale level.

## Data Availability Statement

The data analyzed in this study is subject to the following licenses/restrictions: The raw anonymized imaging data analyzed in this article will be shared for research purpose. Requests to access these datasets should be directed to CL.

## Ethics Statement

The studies involving human participants were reviewed and approved by the First Affiliated Hospital of the Army Medical University Review Board. Written informed consent to participate in this study was provided by the participants’ legal guardian/next of kin.

## Author Contributions

HC mainly focused on patient recruitment, data collection, data analysis, and writing. LD contributed to the genetic sequencing and manuscript revision. YZ, ZJ, and XW collected the clinical, cognitive, and MRI data. LF collected the blood samples. DX contributed to the MRI clinical exclusion and image quality control. JG and HFC contributed to manuscript reviewing and suggestion. JW and CL contributed to the conception, supervision, and reviewing. All authors approved the consent to the attribution of the manuscript.

## Conflict of Interest

The authors declare that the research was conducted in the absence of any commercial or financial relationships that could be construed as a potential conflict of interest.

## Publisher’s Note

All claims expressed in this article are solely those of the authors and do not necessarily represent those of their affiliated organizations, or those of the publisher, the editors and the reviewers. Any product that may be evaluated in this article, or claim that may be made by its manufacturer, is not guaranteed or endorsed by the publisher.

## Footnotes

1
http://www.fil.ion.ucl.ac.uk/spm/software/

## References

[B1] Ahrenfeldt L. J. Lindahl-Jacobsen R. Rizzi S. Thinggaard M. Christensen K. Vaupel J. W. (2018). Comparison of cognitive and physical functioning of europeans in 2004-05 and 2013. *Int. J. Epidemiol.* 47 1518–1528. 10.1093/ije/dyy094 29868871 PMC6208267

[B2] Berman B. D. Smucny J. Wylie K. P. Shelton E. Kronberg E. Leehey M. (2016). Levodopa modulates small-world architecture of functional brain networks in Parkinson’s disease. *Mov. Disord.* 31 1676–1684. 10.1002/mds.26713 27461405 PMC5115928

[B3] Blackburn H. L. Benton A. L. (1957). Revised administration and scoring of the digit span test. *J. Consult. Psychol.* 21 139–143. 10.1037/h0047235 13416432

[B4] Braga-Neto P. Pedroso J. L. Alessi H. Dutra L. A. Felicio A. C. Minett T. (2012). Cerebellar cognitive affective syndrome in machado joseph disease: core clinical features. *Cerebellum* 11 549–556. 10.1007/s12311-011-0318-6 21975858

[B5] Bruno J. Hosseini S. M. Kesler S. (2012). Altered resting state functional brain network topology in chemotherapy-treated breast cancer survivors. *Neurobiol. Dis.* 48 329–338. 10.1016/j.nbd.2012.07.009 22820143 PMC3461109

[B6] Bullmore E. Sporns O. (2009). Complex brain networks: graph theoretical analysis of structural and functional systems. *Nat. Rev. Neurosci.* 10 186–198. 10.1038/nrn2575 19190637

[B7] Chao-Gan Y. Yu-Feng Z. (2010). DPARSF: a matlab toolbox for “Pipeline”. Data analysis of resting-state fMRI. *Front. Syst. Neurosci.* 4:13. 10.3389/fnsys.2010.00013 PMC2889691 20577591

[B8] Folstein M. F. Folstein S. E. McHugh P. R. (1975). MINI-Mental State - practical method for grading cognitive state of patients for clinician. *J. Psychiatric. Res.* 12 189–198. 10.1016/0022-3956(75)90026-6 1202204

[B9] Gamboa O. L. Tagliazucchi E. von Wegner F. Jurcoane A. Wahl M. Laufs H. (2014). Working memory performance of early MS patients correlates inversely with modularity increases in resting state functional connectivity networks. *Neuroimage* 94 385–395. 10.1016/j.neuroimage.2013.12.008 24361662

[B10] Guo J. Chen H. Biswal B. B. Guo X. Zhang H. Dai L. (2020). Gray matter atrophy patterns within the cerebellum-neostriatum-cortical network in SCA3. *Neurology* 95 e3036–e3044. 10.1212/wnl.0000000000010986 33024025

[B11] Hacker C. D. Perlmutter J. S. Criswell S. R. Ances B. M. Snyder A. Z. (2012). Resting state functional connectivity of the striatum in Parkinson’s disease. *Brain* 135(Pt 12), 3699–3711. 10.1093/brain/aws281 23195207 PMC3525055

[B12] Hanssen H. Heldmann M. Prasuhn J. Tronnier V. Rasche D. Diesta C. C. (2018). Basal ganglia and cerebellar pathology in X-linked dystonia-parkinsonism. *Brain* 141 2995–3008. 10.1093/brain/awy222 30169601

[B13] Hernandez-Castillo C. R. Galvez V. Mercadillo R. E. Diaz R. Yescas P. Martinez L. (2015). Functional connectivity changes related to cognitive and motor performance in spinocerebellar ataxia type 2. *Mov. Disord.* 30 1391–1399. 10.1002/mds.26320 26256273

[B14] Hillary F. G. Grafman J. H. (2017). Injured brains and adaptive networks: the benefits and costs of hyperconnectivity. *Trends Cogn. Sci.* 21 385–401. 10.1016/j.tics.2017.03.003 28372878 PMC6664441

[B15] Hosseini S. M. H. Hoeft F. Kesler S. R. (2012). GAT: a graph-theoretical analysis toolbox for analyzing between-group differences in large-scale structural and functional brain networks. *PLoS One* 7:e40709. 10.1371/journal.pone.0040709 22808240 PMC3396592

[B16] Hosseini S. M. H. Kesler S. R. (2013). Comparing connectivity pattern and small-world organization between structural correlation and resting-state networks in healthy adults. *Neuroimage* 78 402–414. 10.1016/j.neuroimage.2013.04.032 23603348 PMC3673883

[B17] Huang S. R. Wu Y. T. Jao C. W. Soong B. W. Lirng J. F. Wu H. M. (2017). CAG repeat length does not associate with the rate of cerebellar degeneration in spinocerebellar ataxia type 3. *Neuroimage Clin.* 13 97–105. 10.1016/j.nicl.2016.11.007 27942452 PMC5133648

[B18] Kawaguchi Y. Okamoto T. Taniwaki M. Aizawa M. Inoue M. Katayama S. (1994). CAG expansions in a novel gene for Machado-joseph disease at chromosome 14q32.1. *Nat. Genet.* 8 221–228. 10.1038/ng1194-221 7874163

[B19] Ko J. H. Spetsieris P. G. Eidelberg D. (2018). Network structure and function in parkinson’s disease. *Cereb. Cortex* 28 4121–4135. 10.1093/cercor/bhx267 29088324 PMC6215468

[B20] Koubiyr I. Besson P. Deloire M. Charre-Morin J. Saubusse A. Tourdias T. (2019). Dynamic modular-level alterations of structural-functional coupling in clinically isolated syndrome. *Brain* 142 3428–3439. 10.1093/brain/awz270 31504228

[B21] Kovacs M. Rush A. J. Beck A. T. Hollon S. D. (1981). Depressed outpatients treated with cognitive therapy or pharmacotherapy. a one-year follow-up. *Arch. Gen. Psychiatry* 38 33–39. 10.1001/archpsyc.1981.01780260035003 7006557

[B22] Li W. Douglas Ward B. Liu X. Chen G. Jones J. L. Antuono P. G. (2015). Disrupted small world topology and modular organisation of functional networks in late-life depression with and without amnestic mild cognitive impairment. *J. Neurol. Neurosurg. Psychiatry* 86 1097–1105. 10.1136/jnnp-2014-309180 25433036 PMC4465874

[B23] Li W. Wang M. Zhu W. Qin Y. Huang Y. Chen X. (2016). Simulating the evolution of functional brain networks in alzheimer’s disease: exploring disease dynamics from the perspective of global activity. *Sci. Rep.* 6:34156. 10.1038/srep34156 27677360 PMC5039719

[B24] Lin F. Wu G. Zhu L. Lei H. (2015). Altered brain functional networks in heavy smokers. *Addict. Biol.* 20 809–819. 10.1111/adb.12155 24962385

[B25] Lucas J. A. Ivnik R. J. Smith G. E. Bohac D. L. Tangalos E. G. Graff-Radford N. R. (1998). Mayo’s older Americans normative studies: category fluency norms. *J. Clin. Exp. Neuropsychol.* 20 194–200. 10.1076/jcen.20.2.194.1173 9777473

[B26] Lyu Y. Guo X. Wang Z. Tong S. (2016). “Resting-state EEG network change in alpha and beta bands after upper limb amputation,” in *Proceedings of the 38th Annual International Conference of the IEEE Engineering in Medicine and Biology Society (EMBC)*, Vol. 8, Orlando, FL, 49–52. 10.1109/EMBC.2016.7590637 28268278

[B27] Meles S. K. Kok J. G. De Jong B. M. Renken R. J. de Vries J. J. Spikman J. M. (2018). The cerebral metabolic topography of spinocerebellar ataxia type 3. *Neuroimage Clin.* 19 90–97. 10.1016/j.nicl.2018.03.038 30035006 PMC6051313

[B28] Meng C. Brandl F. Tahmasian M. Shao J. Manoliu A. Scherr M. (2014). Aberrant topology of striatum’s connectivity is associated with the number of episodes in depression. *Brain* 137(Pt 2), 598–609. 10.1093/brain/awt290 24163276

[B29] O’Callaghan C. Hornberger M. Balsters J. H. Halliday G. M. Lewis S. J. G. Shine J. M. (2016). Cerebellar atrophy in Parkinson’s disease and its implication for network connectivity. *Brain* 139 845–855. 10.1093/brain/awv399 26794597

[B30] Trouillas P. Takayanagi T. Hallett M. Manyarn B. Currier R. D. Subramony S. H. (1997). International cooperative ataxia rating scale for pharmacological assessment of the cerebellar syndrome the ataxia neuropharmacology committee of the world federation of neurology. *J. Neurol. Sci.* 145 205–211. 10.1016/s0022-510x(96)00231-6 9094050

[B31] Peng H. Liang X. Long Z. Chen Z. Shi Y. Xia K. (2019). Gene-Related cerebellar neurodegeneration in SCA3/MJD: a case-controlled imaging-genetic study. *Front. Neurol.* 10:1025. 10.3389/fneur.2019.01025 31616370 PMC6768953

[B32] Pereira J. B. Mijalkov M. Kakaei E. Mecocci P. Vellas B. Tsolaki M. (2016). Disrupted network topology in patients with stable and progressive mild cognitive impairment and alzheimer’s disease. *Cereb. Cortex* 26 3476–3493. 10.1093/cercor/bhw128 27178195 PMC4961019

[B33] Piccinin C. C. Rezende T. J. R. de Paiva J. L. R. Moysés P. C. Martinez A. R. M. Cendes F. (2020). A 5-year longitudinal clinical and magnetic resonance imaging study in spinocerebellar ataxia type 3. *Mov. Disord.* 135 1679–1684. 10.1002/mds.28113 32515873

[B34] Rezende T. J. R. de Paiva J. L. R. Martinez A. R. M. Lopes-Cendes I. Pedroso J. L. Barsottini O. G. P. (2018). Structural signature of SCA3: from presymptomatic to late disease stages. *Ann. Neurol.* 84 401–408. 10.1002/ana.25297 30014526

[B35] Rubinov M. Sporns O. (2010). Complex network measures of brain connectivity: uses and interpretations. *Neuroimage* 52 1059–1069. 10.1016/j.neuroimage.2009.10.003 19819337

[B36] Sanz-Arigita E. J. Schoonheim M. M. Damoiseaux J. S. Rombouts S. A. R. B. Maris E. Barkhof F. (2010). Loss of ‘small-world’ networks in alzheimer’s disease: graph analysis of fmri resting-state functional connectivity. *PLoS One* 5:e13788. 10.1371/journal.pone.0013788 21072180 PMC2967467

[B37] Schmitz-Hubsch T. du Montcel S. T. Baliko L. Berciano J. Boesch S. Depondt C. (2006). Scale for the assessment and rating of ataxia: development of a new clinical scale. *Neurology* 66 1717–1720. 10.1212/01.wnl.0000219042.60538.92 16769946

[B38] Schöls L. Bauer P. Schmidt T. Schulte T. Riess O. (2004). Autosomal dominant cerebellar ataxias: clinical features, genetics, and pathogenesis. *Lancet Neurol.* 5 291–304. 10.1016/S1474-4422(04)00737-9 15099544

[B39] Schoonheim M. M. Hulst H. E. Brandt R. B. Strik M. Wink A. M. Uitdehaag B. M. (2015). Thalamus structure and function determine severity of cognitive impairment in multiple sclerosis. *Neurology* 84 776–783. 10.1212/wnl.0000000000001285 25616483

[B40] Shine J. M. Bell P. T. Matar E. Poldrack R. A. Lewis S. J. G. Halliday G. M. (2019). Dopamine depletion alters macroscopic network dynamics in parkinson’s disease. *Brain* 142 1024–1034. 10.1093/brain/awz034 30887035 PMC6904322

[B41] Silva U. C. A. Marques W. Lourenco C. M. Hallak J. E. C. Osorio F. L. (2015). Psychiatric disorders, spinocerebellar ataxia type 3 and CAG expansion. *J. Neurol.* 262 1777–1779. 10.1007/s00415-015-7807-3 26067219

[B42] Taroni F. DiDonato S. (2004). Pathways to motor incoordination: the inherited ataxias. *Nat. Rev. Neurosci.* 5 641–655. 10.1038/nrn1474 15263894

[B43] Tu Y. Jung M. Gollub R. L. Napadow V. Gerber J. Ortiz A. (2019). Abnormal medial prefrontal cortex functional connectivity and its association with clinical symptoms in chronic low back pain. *Pain* 160 1308–1318. 10.1097/j.pain.0000000000001507 31107712 PMC6530583

[B44] Tzourio-Mazoyer N. Landeau B. Papathanassiou D. Crivello F. Etard O. Delcroix N. (2002). Automated anatomical labeling of activations in SPM using a macroscopic anatomical parcellation of the MNI MRI single-subject brain. *Neuroimage* 15 273–289. 10.1006/nimg.2001.0978 11771995

[B45] Wang Z. Yuan Y. Bai F. You J. Zhang Z. (2016). Altered topolotical patterns of brain networks in remitted late-onset depression: a resting-state fMRI study. *J. Clin. Psychiatry* 77 123–130. 10.4088/JCP.14m09344 26845269

[B46] Watts D. J. Strogatz S. H. (1998). Collective dynamics of ‘small-world’ networks. *Nature* 393 440–442. 10.1038/30918 9623998

[B47] Wu T. Wang L. Hallett M. Chen Y. Li K. Chan P. (2011). Effective connectivity of brain networks during self-initiated movement in Parkinson’s disease. *Neuroimage* 55 204–215. 10.1016/j.neuroimage.2010.11.074 21126588

[B48] Yuan X. Q. Ou R. W. Hou Y. B. Chen X. P. Cao B. Hu X. (2019). Extra-Cerebellar signs and non-motor features in chinese patients with spinocerebellar ataxia type 3. *Front. Neurol.* 10:110. 10.3389/fneur.2019.00110 PMC6388540 30833927

[B49] Zhang J. Wang J. Wu Q. Kuang W. Huang X. He Y. (2011). Disrupted Brain connectivity networks in Drug-Naive. First-Episode Major Depressive Disorder. *Biol. Psychiatry* 70 334–342. 10.1016/j.biopsych.2011.05.018 21791259

[B50] Zhang Y. Qiu T. Yuan X. Zhang J. Wang Y. Zhang N. (2019). Abnormal topological organization of structural covariance networks in amyotrophic lateral sclerosis. *Neuroimage Clin.* 21 101619. 10.1016/j.nicl.2018.101619 30528369 PMC6411656

