# Supplementary material for: Network Reconfiguration Among Cerebellar Visual, and Motor Regions Affects Movement Function in Spinocerebellar Ataxia Type 3
Source: Front Aging Neurosci. 2022 Apr 11;14:773119. doi: 10.3389/fnagi.2022.773119 (PMC9036064; doi:10.3389/fnagi.2022.773119)
Supplement: Supplementary file 1 [file Table_1.DOCX]

**Table S1** **Kolmogorov-Smirnov test value of Data distribution in behavior variables**.

| **Variable** | **Ks** | ***P*** | **Kurtosis** | **Skewness** |
| --- | --- | --- | --- | --- |
| **Onset Age(years)** | 0.068 | 0.992 | 2.401 | 0.253 |
| **Disease duration** | 0.137 | 0.386 | 2.943 | 0.463 |
| **SARA** | 0.159 | 0.229 | 5.066 | 1.375 |
| **ICARS** | 0.189 | 0.094 | 4.029 | 1.037 |
| **ICARS – posture and gait disturbances** | 0.174 | 0.150 | 2.886 | 0.721 |
| **ICARS – kinetic functions** | 0.146 | 0.312 | 5.372 | 1.296 |
| **ICARS dysarthria** | 0.197 | 0.072 | 4.407 | 1.354 |
| **ICARS – oculomotor disorders** | 0.246 | 0.011^*^ | 2.244 | 0.615 |
| **HMAD** | 0.199 | 0.066 | 6.173 | 1.644 |
| **ADL+IADL** | 0.320 | 0.000^***^ | 4.287 | 1.589 |
| **MMSE** | 0.249 | 0.010^**^ | 8.515 | -2.194 |
| **MoCA** | 0.109 | 0.665 | 2.559 | -0.477 |
| **RVR** | 0.137 | 0.383 | 4.230 | 0.851 |
| **DS** | 0.149 | 0.295 | 3.120 | -0.204 |

Values are presented as Kolmogorov-Smirnov test result with ks value, p vale, kurtosis and skewness. Abbreviations: Onset age: Age when patient has ataxia symptoms. Disease duration: Duration between onset age and examination age. SARA: Scale for the assessment and rating of ataxia ICARS: The International Cooperative Ataxia Rating Scale ADL: Activities of daily living. IADL: Instrumental activities of daily living. MMSE: The Mini-Mental State Examination. MoCA: Montreal Cognitive Assessment. RVR: Rapid verbal retrieve. DS: Digit span. HAMD: The Hamilton Rating Scale for Depression.

^*^, P<0.05; ^**^, P<0.01, ^***^, P<0.001.
