# Supplementary material for: Network Reconfiguration Among Cerebellar Visual, and Motor Regions Affects Movement Function in Spinocerebellar Ataxia Type 3
Source: Front Aging Neurosci. 2022 Apr 11;14:773119. doi: 10.3389/fnagi.2022.773119 (PMC9036064; doi:10.3389/fnagi.2022.773119)
Supplement: Supplementary file 2 [file Table_2.docx]

**Table S2 Hub distribution in SCA3 patients and Healthy control group (HCs)**.

| **Hubs only in SCA3 patients** | **Hubs only in HCs** | **Hubs in both groups** |
| --- | --- | --- |
| Right frontal medial gyrus | Right cerebellum Ⅳ_Ⅴ | Left cerebellum Ⅳ_Ⅴ |
| Right frontal superior medial gyrus | Right cerebellum Crus Ⅰ | left cerebellum Ⅵ |
| Right superior gyrus | left anterior cingulum gyrus | left cerebellum crus Ⅰ |
| Right parahippocampal gyrus | Right anterior cingulum gyrus | left cerebellum crus Ⅱ |
| Left rectus gyrus | Left middle cingulum gyrus | Right cerebellum crus Ⅱ |
| Right rectus gyrus | Right lingual gyrus | Left superior frontal gyrus |
|  | Vermis Ⅳ | Left fusiform gyrus |
|  | Vermis Ⅴ | Right fusiform gyrus |
|  | Vermis Ⅵ | Left middle Occipital gyrus |
|  |  | Left middle temporal gyrus |
|  |  | Right middle temporal gyrus |
