# Supplementary material for: Network Reconfiguration Among Cerebellar Visual, and Motor Regions Affects Movement Function in Spinocerebellar Ataxia Type 3
Source: Front Aging Neurosci. 2022 Apr 11;14:773119. doi: 10.3389/fnagi.2022.773119 (PMC9036064; doi:10.3389/fnagi.2022.773119)
Supplement: Supplementary file 3 [file Table_3.docx]

**Table S3 Correlations between clinical symptoms, behavior scales and genetic burden**.

| **Variable** | **CAG repeats** |
| --- | --- |
| **Onset Age(years)** | -0.206 (*0.387*) |
| **Disease duration** | 0.206 (*0.387*) |
| **SARA** | 0.391 (*0.118*) |
| **ICARS** | 0.400 (*0.118*) |
| **ICARS – posture and gait disturbances** | 0.443 (*0.118*) |
| **ICARS – kinetic functions** | 0.337 (*0.158*) |
| **ICARS dysarthria** | 0.271 (*0.276*) |
| **ICARS – oculomotor disorders** | 0.327 (*0.158*) |
| **HMAD** | 0.025 (*0.888*) |
| **ADL+IADL** | 0.127 (*0.543*) |
| **MMSE** | -0.162 (*0.479*) |
| **MoCA** | -0.257 (*0.283*) |
| **RVR** | -0.345 (*0.158*) |
| **DS** | 0.103 (*0.601*) |

Values are presented as correlation coefficient with r ($P_{FDR}$). Abbreviations: Onset age: Age when patient has ataxia symptoms. Disease duration: Duration between onset age and examination age. SARA: Scale for the assessment and rating of ataxia ICARS: The International Cooperative Ataxia Rating Scale ADL: Activities of daily living. IADL: Instrumental activities of daily living. MMSE: The Mini-Mental State Examination. MoCA: Montreal Cognitive Assessment. RVR: Rapid verbal retrieve. DS: Digit span. HAMD: The Hamilton Rating Scale for Depression.
